# Mesoporous Silica-Bioglass Composite Pellets as Bone Drug Delivery System with Mineralization Potential

**DOI:** 10.3390/ijms22094708

**Published:** 2021-04-29

**Authors:** Adrian Szewczyk, Adrianna Skwira, Agnieszka Konopacka, Rafał Sądej, Magdalena Prokopowicz

**Affiliations:** 1 Department of Physical Chemistry, Faculty of Pharmacy, Medical University of Gdańsk, Hallera 107, 80-416 Gdańsk, Poland; adrian.szewczyk@gumed.edu.pl (A.S.); adrianna.skwira@gumed.edu.pl (A.S.); 2 Department of Pharmaceutical Microbiology, Faculty of Pharmacy, Medical University of Gdańsk, Hallera 107, 80-416 Gdańsk, Poland; agnieszka.konopacka@gumed.edu.pl; 3 Department of Molecular Enzymology and Oncology, Intercollegiate Faculty of Biotechnology, University of Gdańsk and Medical University of Gdańsk, Dębinki 1, 80-211 Gdańsk, Poland; rafal.sadej@gumed.edu.pl

**Keywords:** mesoporous silica, bioactive glass, hydroxyapatite, bone regeneration, drug delivery, pellets

## Abstract

For decades, local bone drug delivery systems have been investigated in terms of their application in regenerative medicine. Among them, inorganic polymers based on amorphous silica have been widely explored. In this work, we combined two types of amorphous silica: bioglass and doxycycline-loaded mesoporous silica MCM-41 into the form of spherical granules (pellets) as a bifunctional bone drug delivery system. Both types of silica were obtained in a sol-gel method. The drug adsorption onto the MCM-41 was performed via adsorption from concentrated doxycycline hydrochloride solution. Pellets were obtained on a laboratory scale using the wet granulation-extrusion-spheronization method and investigated in terms of physical properties, drug release, antimicrobial activity against *Staphylococcus aureus*, mineralization properties in simulated body fluid, and cytotoxicity towards human osteoblasts. The obtained pellets were characterized by satisfactory mechanical properties which eliminated the risk of pellets cracking during further investigations. The biphasic drug release from pellets was observed: burst stage (44% of adsorbed drug released within the first day) followed by prolonged release with zero-order kinetics (estimated time of complete drug release was 19 days) with maintained antimicrobial activity. The progressive biomimetic apatite formation on the surface of the pellets was observed. No cytotoxic effect of pellets towards human osteoblasts was noticed.

## 1. Introduction

For decades, amorphous silica has been considered an attractive polymeric material due to its unique properties such as mechanical and thermal resistance, modifiable shape and size of particles, high specific surface area, various chemical functionalization, drug loading capacity, and biocompatibility. Thus, inorganic polymers based on amorphous silica have been widely explored in the pharmaceutical industry as drug delivery systems and biomaterials dedicated to osteomyelitis treatment [1,2]. Osteomyelitis is defined as an inflammation of the bone tissue most often caused by bacterial infection during open bone injuries, bone reconstruction, or implant insertion [3]. The most common treatment of osteomyelitis involves surgery to remove the infected bone fragments, followed by intravenous administration of antibiotics for several weeks [4]. Despite advances in pharmacology and surgery, the bacterial bone infection may remain latent for many years after treatment [5]. That is why increasing interest has been observed for the bifunctional biomaterials which not only release the drug in a prolonged manner directly in the bone site but also support bone regeneration. Such materials represent a promising solution and support the systemic therapy of osteomyelitis, increase patients’ quality of life, and reduce the risk of relapse. Among them, amorphous silica is extensively investigated as a bone regenerative material [6,7].

From a chemical point of view, amorphous silica is a silicon dioxide also known as colloidal, synthetic, or precipitated silica [8]. Presently, amorphous silica dedicated for medical applications is synthesized using a sol-gel process that is facile and effective [9]; however, production of amorphous silica from natural sources has been also explored [10,11]. The sol-gel process is based on two principal reactions: hydrolysis and polycondensation. First, silicon alkoxides, which act as silica precursors, are being hydrolyzed in either acidic or basic media and, in the next step, the obtained products polycondense to form a polymeric network of amorphous silica. Both, the physical and chemical properties of the sol-gel-derived materials are determined by the conditions applied [12]. Undoubtedly, bioactive glasses (bioglasses, BGs) and mesoporous silica materials (MSMs) are the two types of amorphous silica obtained via the sol-gel process which are extremely investigated in terms of pharmaceuticals and medical applications, including treatment of osteomyelitis [13,14].

Bioactive glass powders, composed of SiO_2_-CaO-P_2_O_5_ oxide matrix, were successfully synthesized via a sol-gel process by Li et al. in 1991 [15]. It was confirmed that obtained bioglass could create a surface layer of hydroxyapatite under physiological conditions. Nowadays, such an ability is referred to as mineralization potential. Further in vitro and in vivo studies proved that self-formed a semi-crystalline layer of carbonate apatite on BG surface provides a biocompatible connection between implanted material and bone tissue [16]. Since then, BGs have been widely used in the biomedical area as bone fillers [17], osteoconductive/osteoinductive agent [18], and enamel regenerating material [19]. Unfortunately, conventional BGs are characterized by both low specific surface area and disordered arrangement of pores. Therefore, the adsorption of drug molecules onto the conventional BGs and their use as potential drug delivery systems dedicated to osteomyelitis treatment are limited.

Contrary to BGs, MSMs are characterized by great adsorptive properties due to the large specific surface area, ordered pore structure, uniform pore size distribution, and high surface area to volume ratio. The MSMs were synthesized and described for the first time in 1992 by Charles Kresge’s scientific group [20]. It was claimed that synthesis of ordered structures was possible owing to the presence of surfactant micelles, which allowed inorganic aluminum-silica chains to link around the spherical micelles in an ordered manner. Nowadays, one of the most investigated mesoporous silica materials in regenerative medicine is Mobil Composition of Matter No. 41 (MCM-41) developed by researchers at Mobil Oil Corporation [21]. The MCM-41 material is considered by numerous scientists as a novel bone drug delivery system with potential application in the treatment of bacterial bone infection [22,23,24]. The MCM-41 material offers many advantages such as high drug adsorption capacity, the improvement of drug stability located inside the mesopores channels, prolonged drug release, and increased drug bioavailability directly in bone tissue [25]. Moreover, MCM-41 material has been considered as a biomaterial with potential application in bone repair and orthopedics together with bioglasses and bone cement [26,27]. It was proved that properly modified MCM-41 particles exhibit the mineralization potential; however, the formation of surface apatite is even 30 times longer compared to BGs [6,25]. Importantly, the modification of mesoporous silica material with osteogenic ions may result in disruption of their ordered structure, therefore reproducible and effective drug loading might be limited. Moreover, both BGs and MSMs exist in the form of dusty and adhesive powders, thus their direct use as regenerative materials without proper pharmaceutical processing seems to be unfeasible.

Herein, we aimed to obtain composite-type biomaterial made up of BG and drug-loaded MCM-41. We verified whether these two materials may be successfully combined in a novel composite-type pharmaceutical form that maintains a dual function: drug delivery system and bone tissue regeneration. Both BG and MCM-41 were synthesized using the sol-gel method. Doxycycline hydrochloride (DOX) was chosen as a model antibiotic widely used in the pharmacological treatment of osteomyelitis [28]. The antibiotic was adsorbed onto MCM-41 using a method of adsorption from a concentrated drug solution. Next, both DOX-loaded MCM-41 (MCM-DOX) and BG materials together with excipients (ethylcellulose and polydimethylsiloxane) were combined into the form of spherical granules referred to as pellets. The pharmaceutical form of pellets was chosen due to its desirable properties such as a regular shape, low susceptibility to dose dumping, improved mechanical strength, and flow properties suitable for direct use during surgery. The pellets were investigated in terms of physicochemical properties, drug release, antimicrobial activity, cytotoxicity assay towards human osteoblasts, and mineralization potential in simulated body fluid.

## 2. Results and Discussion

### 2.1. DOX Adsorption onto MCM-41

**Figure 1:**
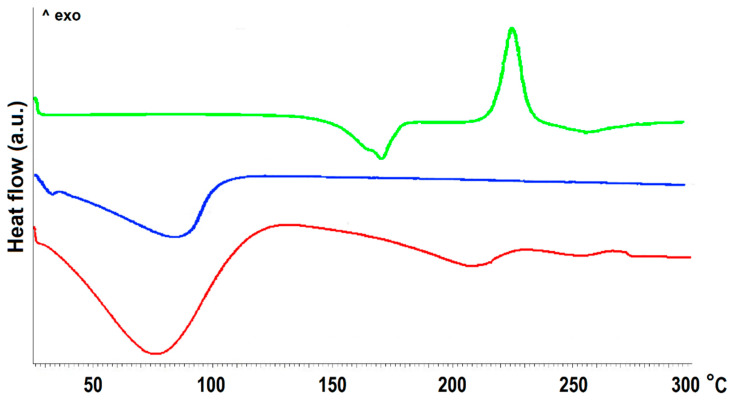
The DSC curves of MCM-41 before (blue) and after (red) DOX adsorption together with DOX reference sample (green).

**Figure 2:**
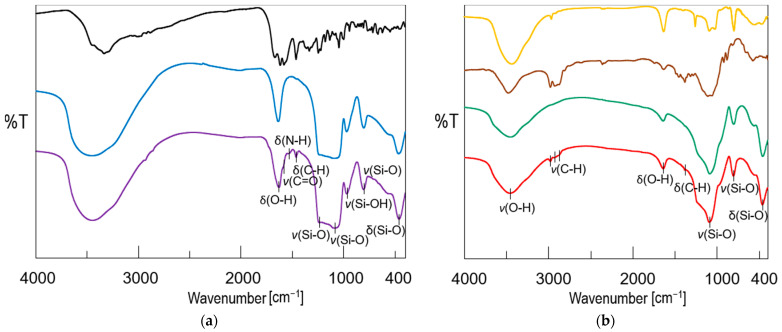
(**a**) FTIR spectra of mesoporous silica MCM-41 before (blue) and after (violet) DOX adsorption together with DOX reference sample (black); (**b**) FTIR spectra of obtained bioglass (green), pellets (red) together with ethyl cellulose (brown), and polydimethylsiloxane (orange) reference samples (types of vibration: *υ*—stretching, *δ*—bending).

The mean amount of doxycycline hydrochloride (DOX) adsorbed onto the MCM-41 was 73.2 ± 1.6 mg per each 1 g of silica material what corresponded to 37.7 ± 0.8% mean adsorption efficiency and 6.8 ± 0.1% drug loading. The reproducible drug adsorption was achieved due to the ordered porous structure of MCM-41 material and is a well-known phenomenon [29,30]. The presence of DOX in the DOX-loaded MCM-41 (MCM-DOX) sample was confirmed by both DSC (Figure 1) and FTIR analyses (Figure 2a).

The differential scanning calorimetry (DSC) curve of the MCM-41 sample revealed only a broad endothermic peak at 80 °C derived from the evaporation of physisorbed water. The curve of the DOX sample was characterized by a sharp endothermic peak at 170.8 °C and an exothermic peak at 225.9 °C attributed to the melting point and thermal decomposition of drug crystals, respectively [31]. In the DSC curve of the MCM-DOX sample, the absence of a sharp peak related to the melting point of DOX might be explained by the incorporation of the drug into the pores of MCM-41 material. Similar findings were reported by Kogawa et al. [32] who investigated the complex structures of doxycycline hyclate with β-cyclodextrin. They have claimed that the absence of the melting peak of doxycycline hyclate in the DSC curve resulted from the formation of an inclusion complex between drug molecules and β-cyclodextrin. Moreover, the disappearance of DSC peaks characteristic for melting points of other drugs such as aprepitant [33], ketoprofen [34], ticagrelor [35] have been reported for various mesoporous silicas and suggested both the successful encapsulation of drug molecules and their amorphous or semi-crystalline nature inside the pores.

The Fourier transform infrared spectroscopy (FTIR) results (Figure 2a) also confirmed the presence of DOX in the MCM-DOX sample and agreed with our previous studies in which DOX was successfully adsorbed onto SBA-15 mesoporous silica [36]. The spectrum of the MCM-DOX showed bands characteristic for both MCM-41 and DOX molecules. The bands at 1631, 1232, 1087, 965, 801, and 462 cm^−1^ derived from MCM-41 (H-O-H deformation vibrations of physisorbed water, external and internal Si-O-Si asymmetric stretching, Si-OH stretching, Si-O-Si symmetric stretching, and O-Si-O deformation vibrations, respectively), whereas the bands at 1580, 1519, and 1458 cm^−1^ were characteristic for DOX molecules (C=O stretching; N-H bending, and C-H bending vibrations, respectively) [36,37].

### 2.2. Physicochemical Characterization of Pellets

The FTIR spectra of obtained pellets, bioglass (BG), and excipients (ethylcellulose—EC, polydimethylsiloxane—PDMS) are compared in Figure 2b. The spectrum of pellets showed the combination of bands characteristic for BG and MCM-41 (H-O-H stretching vibrations at ~3400 cm^−1^ region together with deformation vibrations at 1639 cm^−1^; Si-O-Si stretching vibrations at 1235, 1087, and 802 cm^−1^; O-Si-O bending vibrations at 462 cm^−1^), and ethyl cellulose (C-H stretching vibrations at ~2900 cm^−1^ region; C-H bending vibrations at 1382 cm^−1^). As the BG powder was the main component of pellets formulation (75 wt%), the highest absorption in the spectrum of pellets was observed in the region of bands attributed to BG. No characteristic bands derived from DOX molecules were observed in the FITR spectrum of pellets what suggested that the amount of DOX in the prepared sample was below the detection limit, whereas bands characteristic for PDMS in the region of 1200–400 cm^−1^ were overlapped with BG and MCM-41-derived bands.

**Figure 3:**
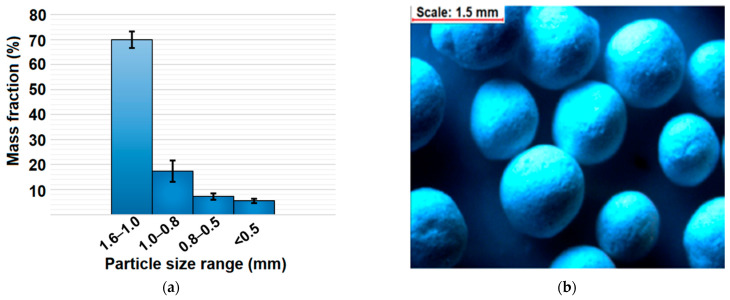
(**a**) Particle size distribution of obtained pellets after sieve analysis; (**b**) stereoscopic view of pellets.

The mean values of pelletization yield, hardness, friability, and drug content of pellets are presented in Table 1, whereas the particle size distribution together with a microscopic view of pellets is presented in Figure 3. The mean yield of the pelletization process was 78 ± 4% providing satisfactory repeatability and efficiency on a laboratory scale (5 g batch size). The obtained pellets were characterized by spherical shape and the main fraction in the range of 1.0–1.6 mm (70 ± 3%) (Figure 3). Such a narrow distribution of pellets provides desirable bulk properties as a consequence of the low plasticity and high dustiness of both MCM-DOX and BG powders. Taking into consideration that obtained pellets were composed of silica materials in the amount > 90 wt%, the obtained particle size distribution was sufficient.

The mean values of pellets hardness and friability were 5.5 ± 1.3 N and 1.1 ± 0.3%, respectively. Based on the obtained results, the pellets were characterized by satisfactory mechanical properties that provided resistance to rough handling during further investigations—no disintegration of pellets was observed during drug release studies and mineralization potential assay. Moreover, the low friability of pellets is considered as one of the factors that provide their mechanical resistance during storage, packing, transport, and other pharmaceutical processes such as coating, compressing, or implanting.

The DOX content in pellets was found to be 91.2 ± 3.5%, thus within the limit given by United States Pharmacopeia for doxycycline tablets (90–120%).

### 2.3. Drug Release Profiles

**Figure 4:**
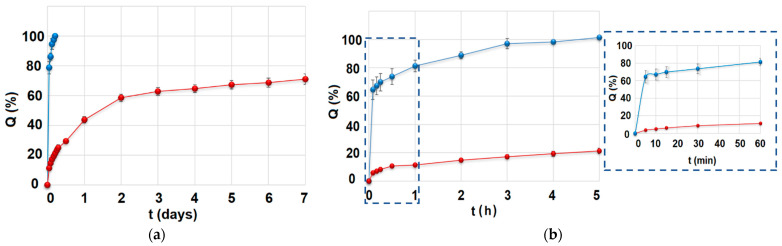
(**a**) The overall drug release profile of MCM-DOX (blue) and pellets (red) samples; (**b**) First 5 h of drug release for both MCM-DOX (blue) and pellets (red) samples with insert related to the first 60 min of drug release.

The drug release profiles of MCM-DOX and pellets are presented in Figure 4. It is observed that MCM-DOX samples were characterized by the complete release of drug within the first 5 h, whereas for pellets a two-stage DOX release was noticed: the initial burst (44 ± 2% after first 24 h) followed by prolonged release. In the case of MCM-DOX samples, the observed burst release was a consequence of DOX high solubility in acceptor fluid. Thus, even the electrostatic interactions and hydrogen bonding between positively charged DOX molecules and negatively charged silica surface cannot impede the dissolution rate of drug particles highly soluble in aqueous media. Moreover, the DOX fraction present on the external silica surface dissolved immediately after immersion in the release medium. A similar release rate of DOX from mesoporous silicas has been previously observed in ours [22,36] and other researchers’ works [38,39].

For the pellets, the reduction in DOX initial release with simultaneous prolongation of total drug release was achieved as a result of the formation of insoluble in the acceptor fluid spherical matrix composed of BG and ethylcellulose used as a filler and binder, respectively. In the case of obtained pellets, the drug release was driven most likely by a gradual penetration of solvent into the pellets matrix causing its partial erosion and increased tortuosity contrary to MCM-DOX powders, for which a classic diffusion seemed to have a predominant impact on DOX release. Moreover, the presumable interactions between DOX molecules and calcium and phosphate ions present in BG might also alter the release rate of the drug. It is known that tetracyclines are characterized by adsorption affinity towards calcium phosphates [40] that may slow down the DOX release rate. However, more experiments must be carried out to verify in detail the potential effect of BG on the DOX release profile.

From a clinical point of view, a two-stage drug release profile characteristic for obtained pellets seems to be a promising feature in current strategies of osteomyelitis treatment using implantable drug delivery systems. Such treatment focuses on both the reduction of systemic dosage and the increase of the antibiotic concentration directly in infected bone [41]. Consequently, a relatively high dose of DOX released during the burst might act as an initial dose already after implantation, whereas the prolonged DOX release might provide the maintenance dose and support bone healing.

The calculated kinetic parameters of DOX release are presented in Table 2. Due to the rapid release of DOX from MCM-DOX samples (>60% in 5 min), the kinetics parameters for these samples are not presented. In our previous studies [22,36], the DOX release from drug-loaded mesoporous silica powders was characterized by simple Fickian diffusion (*n* ≤ 0.43). Herein, when the DOX-loaded MCM-41 powders were transformed into the pellets, the diffusion seemed to be still a predominant mechanism of drug release. Indeed, DOX release profiles of investigated pellets showed a good fit to both the Higuchi (R^2^ = 0.991) and Korsmeyer–Peppas (R^2^ = 0.987) models, suggesting a drug release driven by Fickian diffusion. After the first 24 h of drug release studies, the prolonged release of DOX from pellets was observed following zero-order kinetics (2.4% of dose released per day, R^2^ = 0.972). Based on the zero-order kinetics, the estimated time required for complete drug release was 19 days. Such prolonged release of DOX is a desirable feature of local drug delivery systems for which the release of the drug for sustained periods at a therapeutic level, higher than minimal inhibitory concentrations, has been emphasized [42].

### 2.4. Antimicrobial Activity of Pellets

The CFU/mL values in the function of incubation time of pellets in bacterial medium with corresponding both log reduction and percentage of surviving bacterial cells are presented in Table 3 and Supplementary Figure S1. It was observed that the pellets showed antibacterial activity against *Staphylococcus aureus* compared to control. This observation confirmed that the pellets manufacturing process did not alter the pharmacological activity of the drug. Moreover, the amount investigated pellets equal to 1 mg of DOX exhibited a > 99.9% reduction of CFU/mL providing the minimal bactericidal concentration during 7 days of antimicrobial activity assay. Thus, the antibiotic released from the pellets inhibited the bacterial growth throughout the study what was in accordance with the presented drug release studies (Figure 4). The pellets achieved a mean 4.8-log reduction of bacterial growth compared to control, thus excellent bactericidal activity was proved. Similar log reductions were obtained for cefazolin-loaded pellets in our previous study [43] and for cefazolin/linezolid loaded stainless steel medical grade tubes with potential use as a drug-eluting fixation pin for orthopedic applications [44]. It might be concluded that obtained pellets fulfill an important criterion of bone drug delivery system, where initial high doses of the drug are required for effective local eradication of bacteria, followed by a sustained release which maintains antibacterial effect.

### 2.5. Mineralization Potential Assay

**Figure 5:**
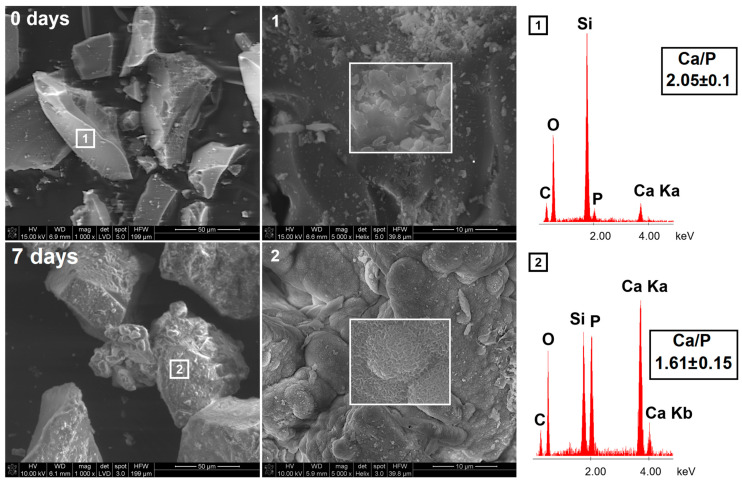
The SEM-EDX micrographs of synthesized bioglass before (upper row) and after (lower row) 7 days of incubation in SBF.

The mineralization potential studies were carried out for both synthesized BG and final pellets. The scanning electron micrographs with energy-dispersive X-ray spectroscopy (SEM-EDX) of BG surface before and after immersion in simulated body fluid (SBF) for 7 days are presented in Figure 5. The obtained BG was characterized by heterogeneous surfaces of flaky-like particles; however, no phase separation was observed. The EDX confirmed the presence of Si, Ca, and P in the sample with a Ca/P molar ratio of 2.05, in agreement with the initial BG composition (theoretical Ca/P = 2.50). After 7 days of incubation in SBF, the surface of BG was completely covered by the hydroxyapatite layer with Ca/P = 1.61, similar to synthetic hydroxyapatite (Ca/P = 1.67) [45]. The observed hydroxyapatite deposits in the form of well-organized spherical clusters exhibited needle-like and cauliflower-like shape characteristics for the biomimetic apatite layer [46].

**Figure 6:**
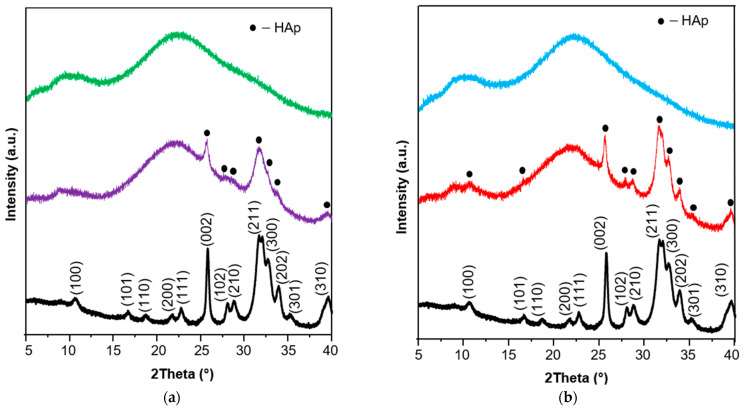
(**a**) The XRD patterns of synthesized bioglass before (green) and after (violet) 7 days of incubation in SBF together with a hydroxyapatite reference sample (black); (**b**) The XRD patterns of pellets before (blue) and after (red) 28 days of incubation in SBF together with a hydroxyapatite reference sample (black).

The surface hydroxyapatite formation on BG particles was also proved using X-ray powder diffraction (XRD) analysis (Figure 6a). For pristine BG, two broad halos in the range of 5–40 2θ revealed the amorphous nature of synthesized material. After the incubation in SBF for 7 days, peaks characteristic for hydroxyapatite (002, 102, 210, 211, 300, 202, 310) appeared, confirming the semi-crystalline nature of the apatite layer [47] formed on the bioglass surface. The high mineralization potential of synthesized BG was proved; therefore, it was used in the manufacturing of pellets as a remineralizing agent.

**Figure 7:**
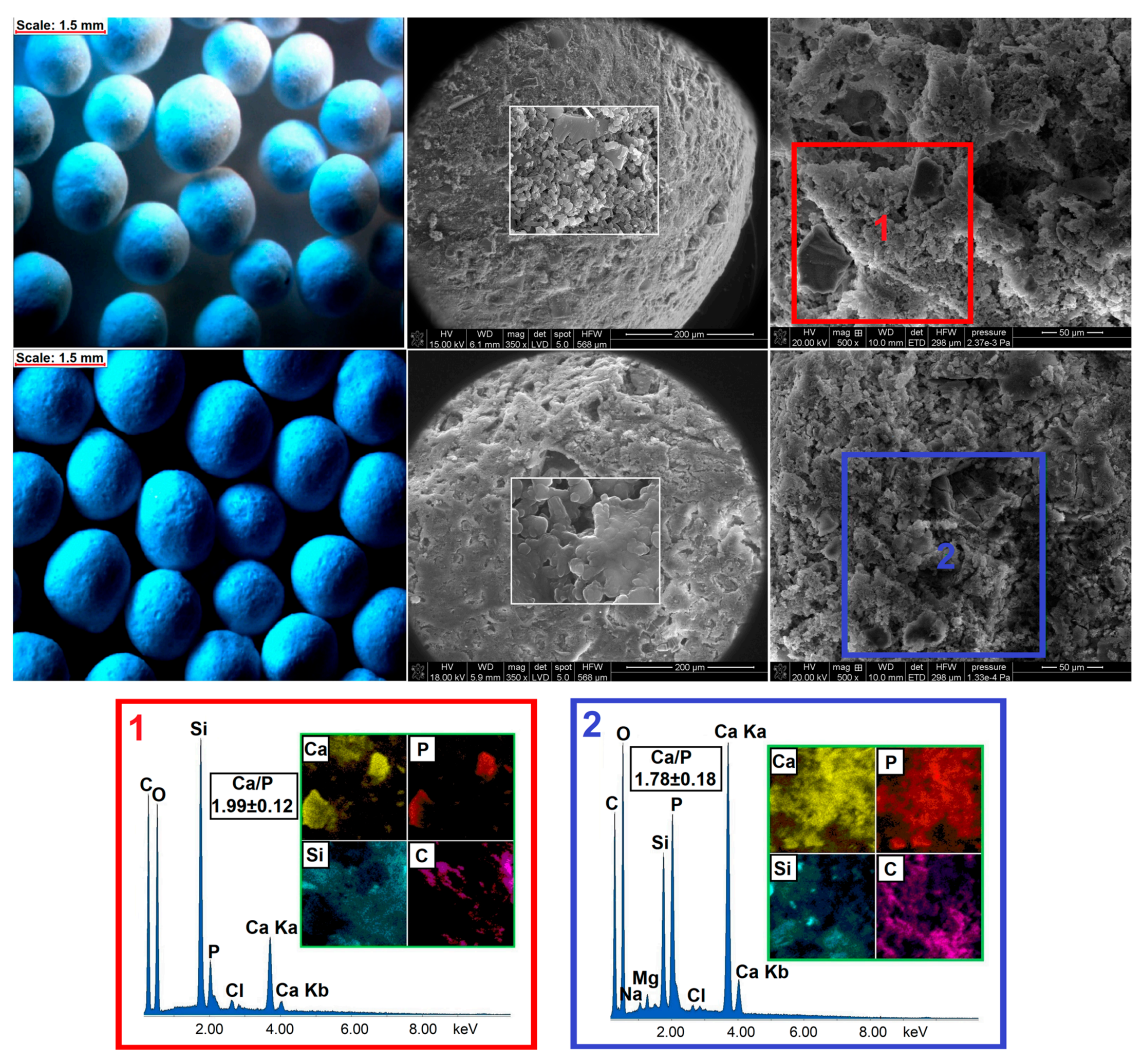
The stereoscopic images and SEM micrographs of pellets before (upper row) and after (lower row) 28 days of incubation in SBF with corresponding EDX mapping.

**Figure 8:**
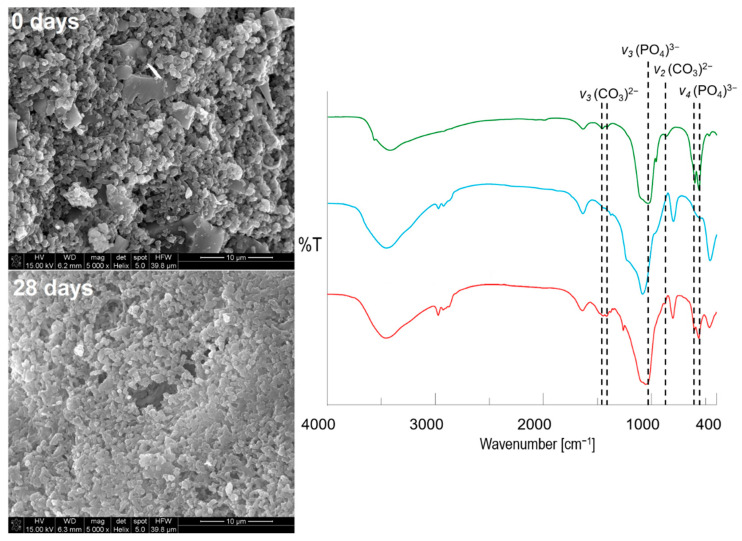
The SEM-EDX micrographs (×5000 magnifications) with corresponding FTIR spectra of obtained pellets before (blue) and after (red) immersion in SBF together with a hydroxyapatite reference sample (green) (types of vibrational modes: *υ*_2_ (bending), *υ*_3_ (stretching) of (CO_3_)^2−^; *υ*_3_ (stretching), *υ*_4_ (bending) of (PO_4_)^3−^.

The hydroxyapatite formation on the surface of pellets was confirmed using SEM-EDX (Figure 7), XRD (Figure 6b), and FTIR (Figure 8) methods. From a stereoscopic point of view, the pellets after incubation in SBF were characterized by small (<100 μm) cavities and holes as a result of their partial erosion caused by SBF penetration (Figure 7). The SEM surface micrographs of pristine pellets showed the spherical particles of DOX-loaded MCM-41 [22], bioglass plates, and fine EC particles. The EDX mapping (Figure 7, red square) revealed three main components of pellets as well—the areas characterized by high Ca and P content, high Si content, or high C content which corresponded to BG, MCM-DOX, and EC, respectively. After 28 days of pellets incubation in SBF, a surface hydroxyapatite layer was observed in the form of randomly packed clusters. With regards to the morphology, formed particles were characterized by spherical and rod-like shapes. As presented in EDX mapping (Figure 7, blue square), both Ca and P spread across the whole investigated area proving the progressive hydroxyapatite formation also confirmed by XRD (Figure 6b). The increased intensities of C suggested that formed hydroxyapatite might be classified as a biomimetic carbonate apatite [48] what is in agreement with FTIR results (Figure 8). The changes in Ca/P molar ratio from 1.99 to 1.78 together with the presence of magnesium and chloride after 28 days of mineralization assay suggested a hydroxyapatite composition similar to the composition of biological bone apatite.

It is well-known that non-modified mesoporous silica MCM-41 is characterized by poor mineralization potential [25], whereas BGs promote hydroxyapatite formation when immersed in SBF. Briefly, the excellent mineralization properties of BG are connected with the presence of osteogenic ions and partial dissolution of silica with simultaneous formation of silanol groups which make the hydroxyapatite nucleation heterogeneous [49]. Most likely, after immersion of BG or pellets in SBF, the solution becomes saturated with the Ca ions released from BG. As mentioned by Gheisari et al. [50], the Ca ions and phosphates can precipitate from concentrated solution either on the surface with already formed apatite nuclei (due to the presence of silanols) or nuclei-free surface. Due to the concentration gradient, Ca ions move preferably toward the apatite nuclei which next grow spontaneously and transform into the continuous layer. Thus, it might be concluded that BG powder added into the pellet formulation acted as a remineralizing agent on which hydroxyapatite nucleation began with nuclei reorganization into the continuous layer of biomimetic apatite. Moreover, the difference in the morphology of apatite formed on BG and pellets surfaces suggests the influence of the presence of mesoporous silica on hydroxyapatite shape. Indeed, the presence of MCM-41 might alter the morphology of formed hydroxyapatite towards nano-size particles [22]; however, this phenomenon will be investigated thoroughly in further studies.

As MCM-DOX particles and formed hydroxyapatite agglomerates were characterized by similar morphology, the SEM images with ×5000 magnifications of pellets surface before and after incubation in SBF together with FTIR spectra are presented in Figure 8. When higher magnification of SEM images was applied, the continuous hydroxyapatite layer on pellets surface was clearly observed after 28 days of mineralization potential assay. The rod-like and partially spherical hydroxyapatite nanoparticles were formed with characteristic densely packaged agglomerates [51]. Contrary to pristine pellets, it was impossible to clearly distinguish both the BG and MCM-41 particles after incubation in SBF, due to the layer of formed apatite which covered all components present on the surface of pellets.

The FTIR spectra of pellets before and after mineralization assay (Figure 8) proved the formation of hydroxyapatite also in agreement with XRD (Figure 6) and SEM-EDX (Figure 7) results.

After incubation of pellets in SBF, an increase in band intensities derived from PO_4_^3−^ groups was observed. Moreover, the shift of band at the maximum peak from ~1080 to ~1040 cm^−1^ confirmed the dominant contribution of hydroxyapatite bonds in the investigated sample. The additional presence of vibrational modes characteristic for CO_3_^2−^ groups provided evidence of carbonates incorporation into the hydroxyapatite structure [52]. Based on SEM-EDX, XRD, and FTIR results it might be concluded that obtained pellets were characterized by the ability to form a carbonate apatite surface layer after immersion in simulated body fluid. It has been emphasized in many studies that such self-formed biomimetic apatite constitutes a completely biocompatible scaffold for further regeneration of the defected bone site after implantation with no need for implant removal surgery [53,54,55].

In our previous works, we investigated different types of bone drug delivery systems with mineralization potential such as xerogels [56,57], membranes [23,29], powders [58,59], granules [55], and pellets [22,43]. In the case of previously described pellets, the mineralization potential was provided by either use of MCM-41-calcium phosphate composite [22] or the addition of hydroxyapatite synthesized via microwave-assisted hydrothermal precipitation method [43]. The difficulty of such pellets manufacturing is the fact that remineralizing component requires the optimization of its synthesis, composition, and the mineralization potential of such component must be confirmed before its use in the formulation of pellets. Herein, the combination of DOX-loaded MCM-41 with bioglass as a remineralizing component seems to overcome the abovementioned difficulties due to the simple synthesis of bioglass and its well-known mineralization potential in vitro and excellent biomineralization in vivo. Moreover, the use of bioglass powder in the pellets formulation allowed to fully replace the microcrystalline cellulose which was used in the previously obtained pellets [22,43]. The elimination of microcrystalline cellulose is another benefit of proposed pellets due to fact that this excipient is characterized by extremely poor mineralization properties.

### 2.6. Cytotoxicity Assay of Pellets

**Figure 9:**
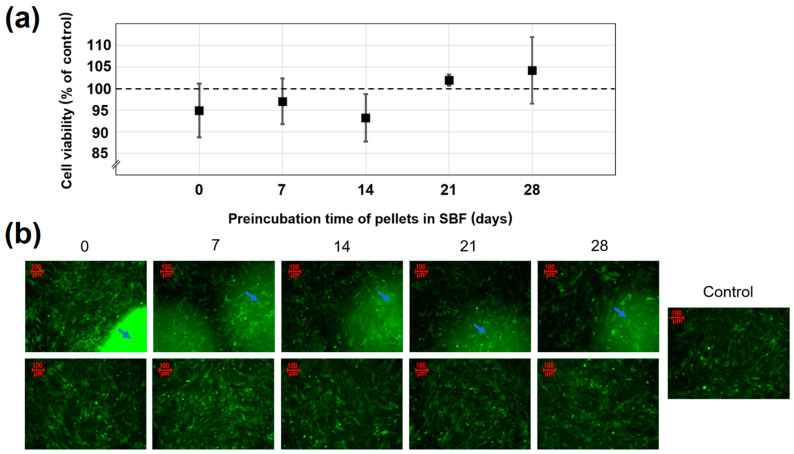
(**a**) The effect of both parent and preincubated in SBF pellets on osteoblast viability; (**b**) The images of the cells before (upper row) and after (lower row) pellets removal. Arrows refer to the areas of osteoblasts directly covered by pellets. Data are represented as mean ± SD (*n* = 3).

The quantitative results of pellets cytotoxicity assay are presented in Figure 9a. In Figure 9b, the representative images in upper and lower rows show the osteoblasts before and after the removal of pellets, respectively. The blue arrows refer to the areas of osteoblasts covered by investigated pellets. The cells cultured with either parent or SBF-preincubated pellets exhibited a typical osteoblastic fusiform shape, identical to the control (cells cultured without any material). There was also no difference in morphology of the cells directly exposed to pellets (arrows in the row A images). The quantitative results confirmed the compatibility of pellets to the human osteoblasts, presenting approximately 100% cell viability after 3 days of incubation. There were no statistically significant differences between the parent and SBF-preincubated cells, which suggests that neither pellets nor apatite formed onto their surface had a visible impact on the osteoblasts proliferation. Moreover, based on the results shown and the stimulatory effect on cell proliferation of low doses of doxycycline previously reported [60,61], it may be claimed that the influence of antibiotic released from the pellets into the culture medium was imperceptible.

## 3. Materials and Methods

### 3.1. Synthesis of Bioglass

The BGs composed of SiO_2_-CaO-P_2_O_5_ were synthesized using a modified molding technique [62,63]. Tetraethyl orthosilicate (TEOS), triethyl phosphate (TEP), and calcium chloride dihydrate (CaCl_2_ × 2H_2_O) were the sources of Si, P, and Ca, respectively (Si/Ca/P = 80:14.4:5.8 molar ratio). All reagents were used as received (reagent grade > 98%, Sigma-Aldrich, Poznan, Poland). First, 10.4 g of TEOS with 4.55 g of purified water and 200 μL of 1 M HCl (POCH, Gliwice, Poland) were mixed in the polypropylene beaker. The beaker was sealed with parafilm and placed on the magnetic stirrer for 30 min (room temperature, 500 rpm) to obtain a homogenous solution. Next, 0.65 g of TEP was added and the mixture was left for 2 h under continuous stirring. Then, 0.99 g of CaCl_2_ × 2H_2_O was added providing an additional 1.5 h of stirring. Finally, the obtained sol was transferred into the porcelain crucible and calcinated at 600 °C for 4 h in a muffle furnace (FCF 7SM, Czylok, Jastrzebie-Zdroj, Poland, 1 °C/min heating rate). Homogenized bioglass powder with a particle size < 100 μm was obtained after fractionation through test sieves (Retsch-Hann, Germany) and used in further investigations.

### 3.2. Synthesis of Mesoporous Silica MCM-41

The synthesis of MCM-41 powders was carried out as described in a previous study [22] using surfactant templating method with TEOS as a silica source and cationic surfactant N-cetyltrimethylammonium bromide (CTAB, reagent grade 99.0%, Sigma-Aldrich, Poznan, Poland) as a template. Briefly, 125 g of water with 12.5 g of ethanol (96% (*v*/*v*) pure, POCH, Gliwice, Poland), 9.18 g of ammonia (25 wt% water solution, POCH, Gliwice, Poland), and 2.39 g of CTAB were stirred together in polypropylene beaker on a magnetic stirrer (300 rpm) for 20 min at room temperature to complete dissolution of surfactant. Next, 10.03 g of TEOS was added drop by drop, and the mixture was left under continuous stirring for 2 h. Then, hydrothermal treatment of the mixture was performed at 90 °C for 5 days without stirring. The resulting powder was recovered by vacuum filtration, washed with 100 mL of pure ethanol, and dried at 40 °C for 1 h. The CTAB template was removed from the solid product using calcination in the air for a period of 6 h at 550 °C (heating rate of 1 °C/min) in a muffle furnace (FCF 7SM, Czylok, Jastrzebie-Zdroj, Poland). The final powders were micronized and 200–500 μm fraction was used in further investigations.

### 3.3. Adsorption of Doxycycline Hydrochloride onto the MCM-41

For the drug-loading experiment, the adsorption procedure was applied as described in our previous reports [64,65]. The MCM-41 material was suspended (1:1 mg/mL ratio) in DOX (Sigma-Aldrich, Poznan, Poland) water solution (10 mg/mL) and incubated in a water bath (25 °C, 300 rpm) for 30 min to ensure the equilibrium adsorption state determined in preliminary studies. Then, materials were centrifuged (9200 rpm, 5 min) and freeze-dried (−52 °C, 0.1 mBar, 24 h). The total amount of DOX adsorbed onto MCM-41 was calculated spectrophotometrically (UV–Vis spectrophotometer Shimadzu, UV-1800, Kyoto, Japan) by monitoring the changes in absorbance at 347 nm and using Equation (1). The adsorption efficiency (*%Ads*) was calculated using Equation (2), whereas the drug loading percentage (*DL*) was calculated using Equation (3). Analytical measurements were conducted following the requirements for quantitative analyses, calibrating the UV-Vis detector with standard solutions of DOX (R^2^ > 0.99).

(1) $$\%Ads=\left(\frac{C_{0}-C_{e}}{C_{0}}\right)\cdot 100\%$$

(2) $$m_{drug}=\left(C_{0}-C_{e}\right)\cdot V$$

(3) $$DL=\left(\frac{m_{drug}}{m_{drug}+m_{MCM-41}}\right)\cdot 100\%$$

where *m_drug_* is an amount of DOX adsorbed at the equilibrium state (mg), *C*_0_ (mg/mL) is an initial DOX concentration, *C_e_* (mg/mL) is DOX concentration at equilibrium state, *V* (mL) is a volume of used DOX solution and *m_MCM_*_-41_ (mg) is the mass of used mesoporous silica material. The MCM-41 powders with adsorbed DOX were named MCM-DOX.

### 3.4. Preparation of Pellets Composed of BG and MCM-DOX

Pellets were obtained on a laboratory scale (5 g batch size) in Caleva Multi Lab apparatus using a preoptimized wet granulation, extrusion, and spheronization method [43]. Silica powders: BG (3.75 g), MCM-41 (0.6 g), and MCM-DOX (0.4 g; equal to 29.3 mg of adsorbed DOX) together with ethyl cellulose (0.25 g, EC, Aqualon N22 Pharm, 20 cP, Warsaw, Poland) were mixed in the mortar and wet-granulated using 4.0 g ethanolic solution (80% (*v*/*v*) of 5 wt% EC with 2 wt% polydimethylsiloxane (PDMS, hydroxy-terminated, 65 cSt, Sigma-Aldrich, Poznan, Poland) as a binder. The excipients used in the pelletization process were pharmacopeia grade and considered non-toxic. Next, the wet mass was extruded in an extruder attachment running at 100 rpm with a circular 1 mm hole diameter and depth. The entire batch of the extrudate was spheronized in a spheronizer attachment with 8.5 cm diameter for 4 min at 2500 rpm. The resultant pellets were left to dry overnight at ambient conditions and stored in a desiccator.

### 3.5. Drug Release Studies

The drug release studies for final pellets were performed using USP II Paddle Apparatus (Copley DIS-6000) at 37.0 °C, 50 rpm. The studies for MCM-DOX powders were also conducted for comparative purposes. 5.3 g of the pellets, 0.8–1.0 mm fraction (equivalent to 30 mg of adsorbed DOX), or 410 mg of MCM-DOX powders (equivalent to 30 mg of adsorbed DOX) were used in tests. A constant fraction of the pellets was used for each batch to minimize the effect of the change in the total surface area of the pellets on the drug dissolution rate. Phosphate buffer solution (pH = 7.4; 500 mL) was used as the dissolution media providing sink conditions. At suitable time intervals, 2.0 mL of solutions were filtered using membrane filters (0.22 μm) and analyzed spectrophotometrically at 347 nm. Analytical studies were conducted in agreement with the requirements for quantitative analyses, calibrating the detector with a standard solution of the tested DOX in phosphate buffer. Drug release data were plotted as the cumulative percent of DOX released (*Q*) as a function of time (*t*). The amounts of DOX released were measured every 24 h for 7 days with 6 replicates.

The parameters of drug release kinetics were calculated using linearized forms of Korsmeyer–Peppas, Higuchi, and zero-order models presented in Equations (4)–(6), respectively:

(4) log Q=n log t+log k

(5) $$Q=k_{H}t^{\frac{1}{2}}$$

(6) $$Q=Q_{0}+k_{0}t$$

where *Q* (%) denotes the fraction released by time *t* (h, day), *n* is an exponent related to the drug release mechanism, *k* (h^−n^) is a rate constant, *k_H_* is a Higuchi dissolution constant (h^−1/2^), *Q*_0_ (%)—the initial fraction of release drug, *k*_0_—zero-order release constant (%/day). For both Korsmeyer–Peppas and Higuchi models, the data for the first 60% of drug release fraction *Q* was fitted.

### 3.6. Antimicrobial Activity Assay

The reference strain of *Staphylococcus aureus* (ATCC 6538) was used in the study as one of the most common bacterial pathogen observed in osteomyelitis [66]. The experiment was carried out using a 12-well titration plate. The weighted pellets (equivalent to 1 mg of DOX) were sterilized with UV rays for 30 min, placed in each well, and flooded with 2 mL of bacterial stock suspension at a density of 10^6^ CFU/mL (Tryptic soy broth medium, TSB, Becton Dickinson, Warsaw, Poland). After 24 h incubation at 37 °C, the suspensions were collected and the amount of survived bacterial colonies (CFU/mL) were determined by serial dilutions in saline—appropriate dilutions were inoculated on the agar plate (Mueller Hinton agar, Becton Dickinson, Warsaw, Poland) for 24 h and counted. As a control, the growth of bacteria without pellets (starting stock suspension) for each day was carried out in the same manner. Simultaneously, the pellets were transferred to a new sterile plate and were flooded again with a freshly-prepared stock suspension of bacteria. The experiment has been conducted for 7 days, in accordance with drug release studies, and was repeated 3 times. The minimal inhibitory concentration of DOX was determined as 4 μg/mL.

### 3.7. Mineralization Potential Assay

The mineralization potential studies were carried out in simulated body fluid (SBF) for both BG powders and final pellets following the procedure proposed by Kokubo and Takadama [67]. Consequently, BG powders were immersed in SBF for 7 days, pellets were immersed for 28 days using a 1:1 (*m*/*v*) or 2:1 (*m*/*v*) ratio between BG powders or pellets and SBF, respectively. Briefly, 100 mg of BG or 200 mg of pellets was immersed in 100 mL of SBF in polypropylene forms. The experiment was performed in a water bath (Witeg WSB-30, Poznan, Poland) at 37.0 °C under stirring conditions (70 rpm). The SBF was exchanged every 24 h using filtration (in the case of BG powders) or by the simple decantation method (in the case of pellets). After 7 or 28 days of incubation in SBF for BG and pellets, respectively; samples were filtered, dried at 40 °C, and analyzed in terms of surface carbonate apatite formation.

### 3.8. Cytotoxicity Assay

Cytotoxicity assay was carried out using the already described procedure [22,43]. Human fetal osteoblasts, hFOB 1.19 (ATCC, cat. no. CRL-11372™), were cultured in a 1:1 mixture of Ham’s F12 Medium Dulbecco’s Modified Eagle’s Medium (DMEM/F12), with 2.5 mM L-glutamine (without phenol red), 15 mM HEPES, and sodium bicarbonate, supplemented with 10% fetal bovine serum and penicillin/streptomycin (100 U/mL/100 μg/mL) at 34 °C in a humidified atmosphere containing 5% CO_2_. The medium was replaced every 2–3 days. Cells were passaged for a maximum of 3–4 months post-resuscitation and regularly tested for mycoplasma contamination. The cytotoxicity of obtained pellets towards human osteoblasts was analyzed by direct contact test, based on the ISO Standard 10993-5: Biological evaluation of medical devices—Part 5: Tests for in vitro cytotoxicity 2009) [68]. This test is related to direct interaction between investigated material and cell monolayer. To avoid false-negative results due to the adsorption of dye (such as MTT, XTT, WST) onto the surface of the pellets, we decided to perform an eGFP fluorescence-based assay which is a reliable and sensitive tool for testing the cytotoxicity of materials [69]. Therefore, at first, osteoblasts were transfected for stable production of enhanced GFP.

To verify the potential influence of the pellets incubation time in SBF on cell viability, pellets preincubated in SBF for 7, 14, 21, and 28 days were examined in parallel with native pellets (not preincubated in SBF). As a control, cells cultured without any material were used. Before the test, pellets were sterilized with UV rays for 30 min. First, cells were seeded in the 96-well plate at a concentration of 8·10^3^ per well. After 24 h of incubation (34 °C, 5% CO_2_), the culture medium from each well was discarded and the pellets were carefully placed onto the cell monolayer. According to ISO Standard 10993-5, investigated material should cover one-tenth of the cell monolayer. Thus, three pellets per one well were tested. Then, the 200 μL of fresh medium was added into each well and incubated for 72 h (34 °C, 5% CO_2_). After the incubation time, the images of the cells cultured with pellets were performed with an Axiovert 200 fluorescent microscope equipped with AxioCam MRm digital camera (Zeiss, Poznan, Poland). Next, pellets were removed from each well, and the conditioned medium was replaced with the fresh one to perform quantitative assessments. Fluorescence intensity was measured with excitation/emission at 485/528 nm using the Synergy H1 microplate reader (BioTek, Winooski, USA). Data were presented as mean value ± standard deviation of 3 independent experiments. Statistical analysis was performed by Student’s t-test using STATISTICA 13.3 software. The results were considered to be statistically significant when the *p*-value was <0.05 vs. control.

### 3.9. Physicochemical Characterization Methods

#### 3.9.1. Fourier Transform Infrared Spectroscopy Analysis

The FTIR measurements were carried out on FT/IR-4700 model (Jasco, Pfungstadt, Germany) using the KBr tablet technique. Each 1 mg of the sample was mixed with 100 mg of KBr, compressed, and analyzed in the range of 4000–400 cm^−1^.

#### 3.9.2. Differential Scanning Calorimetry Analysis

The DSC experiments were carried out using the DSC 822e Mettler Toledo instrument (Warsaw, Poland). Samples of approximately 2–5 mg were placed in a 40 μL aluminum pan with a hole in the lid and analyzed in the range of 25–300 °C (5 °C heating rate) under a nitrogen atmosphere (30 mL/min flow rate).

#### 3.9.3. Powder X-ray Diffraction Analysis

Wide-angle powder XRD data were recorded on the Empyrean PANalytical diffractometer (Malvern, UK) using CuKα radiation (40 kV and 25 mA) at a scanning rate of 1 deg/min with a step width of 0.02 in the 2θ range of 5–40. Prior to analysis, 10 mg of pellets were crushed and homogenized.

#### 3.9.4. Stereoscopic Microscopy

The overall shape of obtained pellets was investigated using stereoscopic microscope Opta-TECH X 2000 (plan-achromatic optic, parallel optical path, Warsaw, Poland).

#### 3.9.5. Scanning Electron Microscopy with Energy Dispersive X-ray Spectroscopy

Surfaces of the samples were examined using SEM-EDX analysis (Hitachi SU-70, Tokyo, Japan and Quanta 3D FEG, Gdansk, Poland). Three samples of pellets and BG powders were independently investigated, choosing five random sites of interest each time. For powders, each sample mass was approx. 5 mg, whereas for pellets each sample included one individual pharmaceutical form. All prepared samples were coated with a 10 nm gold layer and analyzed using 10.0–20.0 kV operating voltage. The Ca/P molar ratio was expressed as a mean value ± SD calculated from the independent EDX measurements.

#### 3.9.6. Yield of Pelletization Process

The yield of the pelletization process was calculated basing on the total weight of dried pellets after pelletization and the initial weight of used excipients. The measurement was carried out for 6 batches and expressed as mean value ± standard deviation.

#### 3.9.7. Size Analysis

The size distribution of pellets was determined using mechanical sieving (HAVER EML 200, Poznan, Poland) with a set of sieves: 0.5, 0.8, 1.0, and 1.6 mm. Pellets of each batch were weighted and shaken for 2 min with an interval of 10 s and an amplitude of 1. The measurement was conducted for 6 batches and expressed as mean values ± standard deviations.

#### 3.9.8. Friability Test

The dust-free pellets (5 g, fraction 0.8–1.0 mm) were weighed, put into the friabilator (Erweka TAR 10, Warsaw, Poland), and rotated for 30 min at 25 rpm. The friability was expressed as a percentage of the weight loss after rotation. The measurement was repeated for 6 batches and expressed as mean value ± standard deviation.

#### 3.9.9. Hardness Test

The Force that causes pellets fracture was determined using the texture analyzer (TA-XTplus, Stable Micro Systems, Torun, Poland). Fifty pellets of each batch (fraction 0.8–1.0 mm) were crushed with the punch which moved down on the pellet with a speed of 1 mm/s. The hardness was measured as a strain corresponding to 50% of the pellet height. The average of the maximum force recorded for fifty individual measurements was reported as the mean hardness of pellets from each batch. The procedure was repeated for 3 batches and expressed as mean value ± standard deviation.

#### 3.9.10. Drug Content Test

The DOX content was examined by completely powdering the 3.0 g of pellets. The obtained powder was suspended in 200 mL of absolute ethanol with vigorous shaking (400 rpm, 24 h, room temperature), providing sink conditions. The total amount of DOX released from the powdered pellets was examined spectrophotometrically. The theoretical 100% content of DOX was calculated including the yield of the pelletization process. The procedure was repeated for 3 batches and expressed as the mean value ± standard deviation.

## 4. Conclusions

In this study, the novel bioglass-mesoporous silica pellets were obtained. The combination of these two components resulted in a bifunctional pharmaceutical form characterized by the biphasic, prolonged release of doxycycline and simultaneous induction of hydroxyapatite formation under in vitro conditions. Preliminary microbiological and biological analyses showed that the obtained pellets maintained active against *Staphylococcus aureus* throughout the total incubation time with no harmful effect on human osteoblast. It is noteworthy that the pellets remained mechanically resistant during the investigations in which various, sometimes rigorous, conditions were applied. Moreover, contrary to dusty silica powders, the final form of pellets may significantly facilitate materials intraoperative implantation. Nonetheless, due to the specific limitations of the in vitro studies, further investigations that include animal models must be carried out to verify whether the obtained pellets may not only have the potential ability to support antibacterial treatment as a local drug delivery system but also may initiate regeneration of affected bone tissue. As the interface between material surface and bone tissue is the area of complex biological interaction, further investigations of the impact of the pellets on osteoblast differentiation will be performed in the next step.

## Figures and Tables

**Table 1:** The physical characteristic of obtained pellets together with the yield of the pelletization process.

Yield (%)	Hardness (N)	Friability (%)	Drug Content (%)
78 ± 3	5.5 ± 1.3	1.1 ± 0.3	91.2 ± 3.5

**Table 2:** The calculated kinetic parameters of DOX release for obtained pellets.

Higuchi Model		Korsmeyer–Peppas Model		Zero-Order Kinetics *	
k_H_	R^2^	*n*	R^2^	k_0_	R^2^
7.3	0.991	0.36	0.987	2.4	0.972

R^2^—coefficient of determination, k_H_—Higuchi dissolution constant (h^−1/2^), *n*—release exponent of Korsmeyer–Peppas model, k_0_—zero-order release constant (% of dose released per day). * calculated excluding first 24 h of DOX release.

**Table 3:** The calculated mean values of bacterial concentration (CFU/mL) of control and bacterial culture media were incubated with pellets together with log difference and percentage of killed bacteria after antimicrobial activity studies.

Day	CFU/mL		Log_10_ Difference	Percent of Killed Bacteria
	Control	Pellets		
1	1.4 × 10^9^	1.4 × 10^5^	4.0	>99.9%
2	1.8 × 10^9^	6.2 × 10^3^	5.5	
3	4.7 × 10^9^	4.5 × 10^4^	5.0	
4	5.4 × 10^9^	8.0 × 10^4^	4.8	
5	6.5 × 10^9^	1.5 × 10^5^	4.6	
6	2.0 × 10^10^	1.0 × 10^5^	5.3	
7	1.0 × 10^10^	3.5 × 10^5^	4.5	

max. SD for obtained results: ±7%.

## Data Availability

The data presented in this study are available on request from the corresponding author.

## Supplementary Materials

The following are available online at https://www.mdpi.com/article/10.3390/ijms22094708/s1, Figure S1: The obtained CFU/mL value as a function of time for both bacterial culture media: incubated with pellets (red) and control (blue).
