# Supplementary material for: Mesoporous Silica-Bioglass Composite Pellets as Bone Drug Delivery System with Mineralization Potential
Source: Int J Mol Sci. 2021 Apr 29;22(9):4708. doi: 10.3390/ijms22094708 (PMC8124432; doi:10.3390/ijms22094708)
Supplement: Supplementary file 1 [file ijms-22-04708-s001.zip › ijms-1186322-supplementary.pdf]

International Journal of *Molecular Sciences*

MDPI

Article

Mesoporous silica-bioglass composite pellets as bone drug delivery system with mineralization potential

Adrian Szewczyk [1], Adrianna Skwira [1], Agnieszka Konopacka [2], Rafał Sądej [3] and Magdalena Prokopowicz [1,*]

[1] Department of Physical Chemistry, Faculty of Pharmacy, Medical University of Gdańsk, Hallera 107, 80-416 Gdańsk, Poland; adrian.szewczyk@gumed.edu.pl; adrianna.skwira@gumed.edu.pl
[2] Department of Pharmaceutical Microbiology, Faculty of Pharmacy, Medical University of Gdańsk, Hallera 107, 80-416 Gdańsk, Poland; agnieszka.konopacka@gumed.edu.pl
[3] Department of Molecular Enzymology and Oncology, Intercollegiate Faculty of Biotechnology, University of Gdańsk and Medical University of Gdańsk, Dębinki 1, 80-211 Gdańsk, Poland; rafal.sadej@gumed.edu.pl
* Correspondence: magdalena.prokopowicz@gumed.edu.pl

Supplementary material

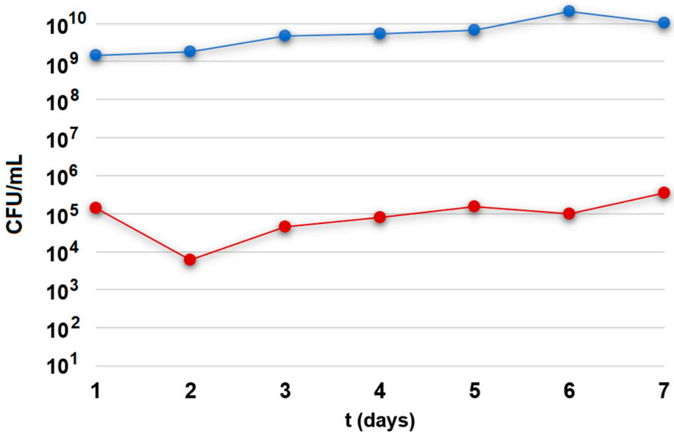

Figure S1. The obtained CFU/mL value as a function of time for both bacterial culture media: incubated with pellets (red) and control (blue).
